# Brain Matters in Duchenne Muscular Dystrophy: DMD Mutation Sites and Their Association with Neurological Comorbidities Through Isoform Impairment

**DOI:** 10.3390/genes17010012

**Published:** 2025-12-24

**Authors:** Teodora Barbarii, Raluca Anca Tudorache, Dana Craiu, Elena Neagu, Lacramioara Aurelia Brinduse, Carmen Magdalena Burloiu, Catrinel Mihaela Iliescu, Magdalena Budisteanu, Ioana Minciu, Diana Gabriela Barca, Carmen Sandu, Oana Tarta-Arsene, Cristina Pomeran, Cristina Motoescu, Alice Dica, Cristina Anghelescu, Dana Surlica, Adrian Ioan Toma, Niculina Butoianu

**Affiliations:** 1 Department of Medical Genetics, “Carol Davila” University of Medicine and Pharmacy, 020032 Bucharest, Romania; barbariiteodora@gmail.com; 2 Romanian Group for Undiagnosed Rare Diseases, Genomics Research and Development Institute, 020021 Bucharest, Romania; 3 Pediatric Neurology Discipline, Department of Neurosciences, “Carol Davila” University of Medicine and Pharmacy, 050474 Bucharest, Romania; 4 Université Paris Cité, 75015 Paris, France; 5 Pediatric Neurology Clinic, “Prof. Dr. Alexandru Obregia” Clinical Hospital, Expertise Center for Rare Pediatric Neurology Disorders, EpiCARE Full Member, 041914 Bucharest, Romania; 6 Dr. Nicolae Robanescu National Clinical Center for Children Neurorehabilitation, 041408 Bucharest, Romania; elenaneagu1097@gmail.com; 7 Public Health and Management Department, “Carol Davila” University of Medicine and Pharmacy, 050463 Bucharest, Romania; 8 Research Department for Psychiatry, “Prof. Dr. Alexandru Obregia” Clinical Hospital, 041914 Bucharest, Romania; magda_efrim@yahoo.com; 9 Medical Genetics Laboratory, Victor Babes National Institute of Pathology, 050096 Bucharest, Romania; 10 Department of Genetics, Faculty of Medicine, “Titu Maiorescu” University, 040051 Bucharest, Romania; 11 Ioana Medical Center, 011053 Bucharest, Romania; 12 Faculty of Medicine, “Titu Maiorescu” University, 040051 Bucharest, Romania

**Keywords:** duchenne muscular dystrophy, DMD gene, dystrophin isoforms, brain comorbidities, Dp140 5′ UTR variants, functional classification

## Abstract

Background: Duchenne/Becker muscular dystrophy (DMD/BMD) is associated with a wide spectrum of brain-related comorbidities. Methods: This retrospective study assesses the neuropsychiatric profile of DMD/BMD patients and the hypothesis of a functional-versus-structural approach of dystrophin gene variants/impaired isoforms in relation to brain comorbidities. Patients with documented mutation in the DMD gene and neuropsychiatric assessments were included. Seven comorbidities were analyzed based on variant location and dystrophin brain isoform disruption. The clustering of comorbidities and genotype–phenotype correlations were studied. Results: 264 DMD/BMD patients met inclusion criteria. 22 variants have never been described before. A high prevalence of neuropsychiatric comorbidities was identified in the cohort with higher values in patients with distal mutations. The number of comorbidities increased with the number of brain dystrophin isoforms predicted to be lost. Functional-versus-structural comparison revealed that Dp140 5′UTR variants might not affect protein expression. Epilepsy and intellectual disability (ID) showed significant association in this cohort. Neuropsychiatric phenotype varied greatly in patients with identical variants, even between siblings. Conclusions: This is one of the largest European cohorts for which all these comorbidities were studied in association with DMD gene mutation site and the first study of this kind performed on the Eastern European DMD/BMD population. Our group analyzed, for the first time, Dp140 5′UTR variants in relation to all neuropsychiatric phenotypes and showed that epilepsy and ID are strongly associated in DMD/DMB patients.

## 1. Introduction

Duchenne/Becker Muscular Dystrophy (DMD/BMD) is a progressive neuromuscular condition caused by mutations in the dystrophin gene (DMD/Xp21) resulting in the lack of functional dystrophin protein [1]. Dystrophin protein’s role is not limited to muscle function as there is increasing evidence of neurological, psychiatric, and neurodevelopmental comorbidities associated with impaired dystrophin isoforms expressed in different brain areas [2,3,4]. The French neurologist Duchenne de Boulogne, who initially described the disease, noted the presence of cognitive deficits as part of the phenotype [5] and it was reported that global developmental delay and cognitive and language impairments could be the initial symptoms of the disease [6].

**Figure 1:**
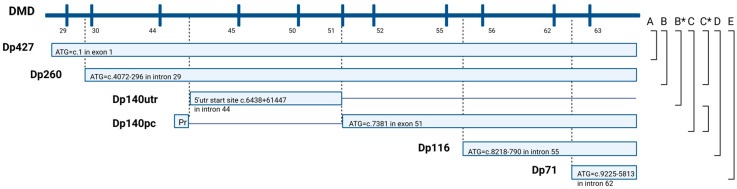
DMD gene, dystrophin isoforms, and patients groups. Vertical lines represent the exons of the DMD gene. Dp427, Dp260, Dp140, Dp116, and Dp71 are dystrophin isoforms. Transcription of every isoform is initiated at ATG (Methionine) and the location of ATG is listed for all isoforms transcripts. The promoters of isoforms are localized prior to these transcription initiation sites. Dp140 transcript includes a long 5′utr (untranslated region)-Dp140utr consisting of exons 45–50 and a portion of exon 51 and has its promoter (Pr) within intron 44, followed by the protein coding region of the isoform (Dp140pc). From the N- to C-terminus, dystrophin contains an N-terminal actin-binding domain, a central rod domain made of spectrin-like repeats, a cysteine-rich domain, and a C-terminal domain. Dp427 (3685 aa) contains all four domains; Dp260 (2344 aa) lacks the N-terminal actin-binding domain; Dp140 (1225 aa) lacks the N-terminal domain and part of the rod domain; Dp116 (956 aa) contains only the last rod repeats plus the cysteine-rich and C-terminal domains; and Dp71 (617 aa) consists mainly of the cysteine-rich and C-terminal domains with a short unique N-terminus. Structural (A–E) and functional (B*–C*) patient groups, according to mutation position and predicted isoform disruption, are indicated on the right and described in detail in Section 2.3. This figure is adapted from Taylor et al. [21] with permission. * = functional groups. utr/UTR = untranslated region.

The dystrophin gene, one of the largest and complex genes in the human genome, has 79 exons and integrates 7 promoters, giving rise to 7 dystrophin protein (Dp) isoforms with diverse expression in different tissues [7,8]. Variants at the 5′ end of the gene affect the longest isoforms generated by the three promoters located upstream the first exon, named Dp427M, Dp427C, and Dp427P, according to the main sites of their expression: skeletal and cardiac muscle, neurons of the cerebral cortex, and hippocampus and cerebellar Purkinje cells, respectively [9,10,11,12]. Mutations occurring further downstream of the gene affect, besides the long isoforms, the other gene products deriving from the four internal promoters located distal to exon 30. Variants between exons 31 and 44 disrupt the isoform Dp260 (expressed predominantly in the retina [13]), those between exons 45 and 62 affect Dp140 (expressed at high levels in the brain during fetal development and adult kidneys [14,15]) and Dp116 (expressed in adult peripheral nerves [16]), and mutations occurring downstream of exon 63 affect all dystrophin gene products including isoform Dp71 (the most abundant isoform expressed in the brain, even from fetal life [17]) (Figure 1).

Many studies investigated the relationship between neuropsychiatric comorbidities and gene mutation sites in DMD patients. Ricotti and colleagues [18] were the first to observe a pattern when clustering these brain-related symptoms and proposed the term “DMD neuropsychiatric syndrome”. Several studies pointed out that mutations in the second part of the dystrophin gene are associated with cognitive impairment and neuropsychiatric comorbidities as they affect more dystrophin gene products. Therefore, patients with 3′ end variants present a more severe neurodevelopmental phenotype following the cumulative loss of brain isoforms [19,20,21,22].

Compared with the general pediatric population, DMD/BMD patients have a higher prevalence of intellectual disability (ID) [23] and other neuropsychiatric comorbidities, including attention-deficit/hyperactivity disorder (ADHD) [24,25], autism spectrum disorders (ASD) [26,27], obsessive–compulsive disorder, depression, aggression, anxiety, motor and vocal tics [28], and epilepsy [29,30,31,32].

## 2. Materials and Methods

### 2.1. Patients

This is a single-center retrospective observational study on a cohort of DMD/BMD patients referred for assessment at the Pediatric Neurology Department, Prof. Dr. Alex. Obregia Clinical Hospital of Psychiatry, Bucharest, Romania, between January 2001 and July 2024.

Inclusion criteria were as follows: 1. males with DMD/BMD clinical diagnosis; 2. confirmed molecular diagnostic in the dystrophin gene; 3. age 2–20 years; 4. documented neurological, psychiatric, and psychological assessment; and 5. absence of severe hearing or visual impairment. The exclusion criteria were as follows: 1. female carriers (symptomatic females were not included since skewed X-chromosome inactivation produces tissue-specific mosaic dystrophin expression, making their brain isoform profile not directly comparable with that of hemizygous males); 2. patients missing neurological, psychiatric, and psychological assessment; and 3. other neurological disorders than DMD.

This study was approved by the Ethics Committee of Prof. Dr. Alexandru Obregia Clinical Hospital of Psychiatry according to the Declaration of Helsinki. Informed consent for molecular testing and clinical assessments, as well as for results publication, is included in the standard informed consent documents of the hospital.

### 2.2. Clinical Assessment

The clinical data were extracted from patients’ records. The patients’ care was routinely performed according to the international guidelines (standard of care) by a multidisciplinary team [33,34].

The following domains were evaluated:

1. The neurodevelopmental disorders (NDDs) including intellectual disability (ID), attention-deficit/hyperactivity disorder (ADHD), autism spectrum disorder (ASD), and language and/or speech disorders (LSD). Cognitive ability was measured using Wechsler Intelligence Scales for Children (WISC), Raven’s Progressive Matrices, or Mental Developmental Scales depending on the patient’s age. Patients were divided into intellectually normal or disabled, based either on the IQ or DQ at the moment of the assessment. A small number of patients had severe developmental delay and were not able to complete these scales therefore they were directly categorized into the intellectual disability group. The diagnosis of ADHD and ASD was established by pediatric psychiatrists based on criteria of the Diagnostic and Statistical Manual of Mental Disorders (DSM IV-TR and DSM V). Patients were classified as having language and/or speech disorders based on Verbal Comprehension Index scores from the Wechsler Intelligence Scales for Children (WISC) and based on the healthcare professionals’ reports at presentation.
2. The behavioral emotional (BE) group was divided in two: a. the externalizing symptoms (ES) group including oppositional/aggressive behavior, anger problems, and behavioral outbursts—analyzed by pediatric neurologists at presentation or reported by the family; and b. the internalizing symptoms (IS) group including anxiety and depression—diagnosis was established by pediatric psychiatrists based on the criteria of the DSM IV-TR/DSM V.
3. The diagnosis of epilepsy was established based on history, clinical aspects of seizures, and EEG evaluation findings. Seizure diagnosis respected the International League Against Epilepsy (ILAE) classification [35,36,37]. Patients with febrile seizures, family history of epilepsy, or other known etiology for the seizures were excluded from this category.

### 2.3. Genetic Analysis

Genomic DNA was extracted from peripheral blood samples. DMD exon-level deletions and duplications were first screened using SALSA MLPA probemixes P034 and P035 (MRC-Holland, Amsterdam, The Netherlands), which cover all exons of the DMD gene. In patients without a copy-number variant detectable by MLPA, the entire coding region and exon–intron boundaries of the DMD gene were analyzed either by Sanger sequencing or, in more recent years, by next-generation sequencing approaches (targeted neuromuscular panels, exome or genome sequencing) to detect indels, point mutations, and splice-site variants. Sequence variants identified by NGS were confirmed by Sanger sequencing where necessary. Variants were classified according to the ACMG/AMP classification guidelines [38,39].

Testing protocols were carried out in several laboratories. Most MLPA and Sanger sequencing analyses were performed in Romanian diagnostic laboratories starting with 2005, and expenses were supported by the Romanian Ministry of Health through different National Disease Prevention Programs. Next-generation sequencing was performed in reference laboratories abroad. The patients diagnosed clinically and by immunohistochemistry before 2005 were also diagnosed by molecular investigations.

Patients were divided into different groups according to the location of their mutation and the affected dystrophin isoforms, as presented by Taylor et al. [21] (Figure 1). A detailed distribution of neuropsychiatric comorbidities prevalence was studied using the following structural and functional classifications:

#### 2.3.1. Structural Classification of the Identified DMD Variants

The structural classification was based only on the location of the variant (prior to or after a specific exon). Therefore, patients were divided in the following 5 groups: group A—variants located upstream of exon 30 which are considered to affect Dp427m, Dp427c, and Dp427p isoforms; group B—variants between exons 31 and 44, affecting Dp427 and Dp260; group C—variants between exons 45 and 55, affecting Dp427, Dp260, and Dp140; group D—variants between exons 56 and 62, affecting Dp427, Dp260, Dp140, and Dp116; group E—variants located downstream exon 63, affecting all dystrophin isoforms including Dp71 (Figure 1).

#### 2.3.2. Functional Classification of the Identified DMD Variants

This classification consisted of selecting and grouping patients based on the isoforms disrupted. We consider that the disruption of an isoform is caused by a mutation either located on the protein coding region of the isoform or affecting the isoform’s promoter (deletion of the promoter).

We excluded patients carrying variants for which the functional effect cannot be predicted: large duplications containing internal promoters and large deletions and duplications with proximal breakpoints at the border of internal promoters (e.g., deletion of exon 44 or exon 45 detected by MLPA may or may not extend to the Dp140 promoter; therefore, we cannot be certain whether Dp140 expression is preserved or lost in these patients). The location of every promoter is displayed in Figure 1.

The selected patients were included in the following functional groups: group A*—variants affecting Dp427m, Dp427c, and Dp427p isoforms (located upstream of exon 29); group B*—variants affecting Dp427, Dp260 (located between exons 30 and 43) and Dp140utr (mutations between exons 46 and 50, that do not affect in theory the Dp140 isoform); group C*—variants affecting Dp427, Dp260, and Dp140 (located between exons 51 and 54 affecting Dp140pc and those affecting the Dp140 promoter, e.g., deletion of exons 44–45); group D*—variants affecting Dp427, Dp260, Dp140, and Dp116 isoforms (located between exons 56 and 61); group E*—variants affecting all dystrophin isoforms including Dp71 (located downstream exon 63) (Figure 1).

An additional group Y comprising patients with large deletion breakpoints at the border of the Dp116 promoter was created (e.g., deletion of exons 51–55). It is not certain whether mutations from this group, besides disrupting Dp427 and Dp140 isoforms, also affect Dp116. However, these patients can be included in the statistical analysis when grouping C* with D* (C* + Y + D*) since, on a functional level, patients from the Y group can belong to either C* or D*.

#### 2.3.3. Functional Versus Structural Comparison and Study of Dp140 5′ UTR Variants

As a certain number of patients were excluded for the functional classification (see Section 2.3.2), the comparison had to be performed using a smaller cohort of patients. The patients selected for the functional classification were divided into structural groups using the same criteria as in Section 2.3.1. Thus, smaller structural groups were generated and annotated as follows: group A’, B’, C’, D’ and E’.

**Figure 2:**
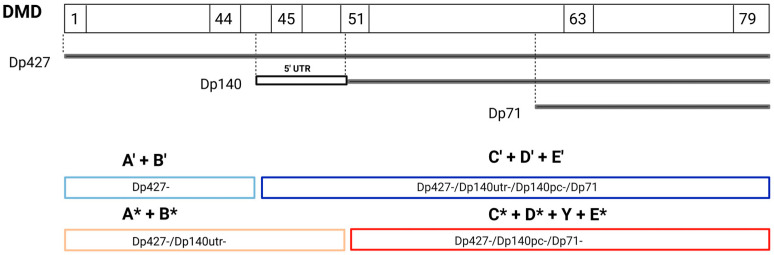
DMD gene, dystrophin brain isoforms, and patients groups according to structural (blue) and functional (red) classification applied for the cohort of 203 patients. Light blue: loss of Dp427 isoform (patients with variants upstream exon 44—structural groups A’ + B’), dark blue: loss of all brain isoforms (patients with variants downstream exon 45—structural groups C’ + D’ + E’), light red: loss of Dp427 (patients with variants upstream exon 44 and mutations in the Dp140 5′ UTR region—functional groups A* + B*), dark red: loss of all brain isoforms (patient with variants downstream exon 51—functional groups C* + D* + Y + E*). * = functional groups.

To study the effect of Dp140 5′ UTR variants, we compared 2 possible scenarios: 5′ UTR variants affecting Dp140 expression-patients with mutations in the 5′ UTR region are grouped distally in group C’ (structural classification) versus 5′ UTR variants that do not affect Dp140 expression–patients with mutations in the 5′ UTR region are grouped proximally in group B* (functional classification): A’ + B’/C’ + D’ + E’ versus A* + B*/C* + D* + Y + E* (Figure 2).

#### 2.3.4. Genotype–Phenotype Correlations

Phenotypes of individuals carrying the same variant were compared. Only mutations affecting certain dystrophin isoforms (the functional approach) and those found in siblings were studied.

### 2.4. Statistical Analysis

Statistical analyses were performed using SPSS version 23.0 and R version 4.5.2. For each feature, Chi-square test or Fisher’s exact test were used to compare the frequencies between the groups of patients classified according to specific classifications. A Chi-square test was used to assess the clustering of symptoms based on genotype groups. The correlation between the number of affected brain isoforms and the number of neuropsychiatric comorbidities was assessed using Spearman’s rank correlation coefficient. Kendall rank correlation coefficient was used to assess the association between comorbidities. *p*-values less than 0.05 were considered statistically significant.

## 3. Results

264 boys with DMD/BMD with an age range of 2 to 20 years were enrolled for more than 23 years. 239 (90.5%) patients had DMD and 25 had the (9.4%) BMD phenotype. 11 pairs of brothers were identified. All patients were classified in different groups based on mutation location or affected dystrophin isoforms.

### 3.1. Genetic Results

The following variants were detected: 163 (61.7%) large deletions and 30 (11.3%) large duplications, 37 (14%) nonsense, 6 (2.2%) missense, 21 (7.9%) small insertions or deletions and 5 (1.8%) splice site mutations. 22 variants have never been reported before (absent from LOVD, ClinVar and HGMD databases). In two patients, only the exon where the premature stop codon was located was specified. All the variants and associated phenotypes described in this paper were submitted to the LOVD (https://www.LOVD.nl/DMD) and are listed in Supplementary Table S1.

### 3.2. Structural Classification of the Identified DMD Variants

According to the structural classification, the 264 patients were distributed as follows: group A—72 (27.2%) patients, group B—37 (14%), group C—122 (46.2%), group D—12 (4.5%) and group E—21 (7.9%).

The cohort of 264 patients presented the following neurodevelopmental features: ID—82 (31.1%) patients, ADHD—63 (23.9%), ASD—19 (7.2%), and LSD—61 (23.1%). ES were present in 16 (6.1%) males whereas IS were found in only 7 (2.7%) patients. Epilepsy was present in 14 (5.3%) patients (Supplementary Table S2).

**Figure 3:**
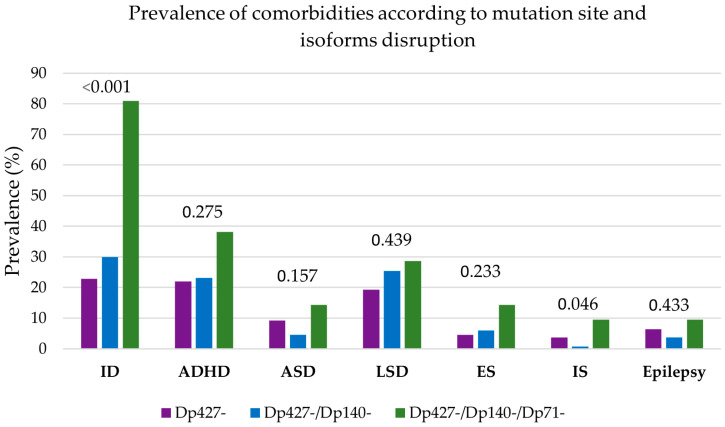
Prevalence of neuropsychiatric comorbidities for the cohort of 264 patients according to the structural classification. Purple: loss of Dp427 isoform (groups A + B), blue: loss of Dp427 and Dp140 isoforms (groups C + D), green: loss of Dp427, Dp140 and Dp71 isoforms (group E). For each comorbidity, the *p*-value of the global test for prevalence differences among the three isoform-loss groups (Chi-square or Fisher’s exact test) is indicated above the corresponding bars. ADHD = attention deficit hyperactive disorder, ASD = autism spectrum disorder, ES = externalizing symptoms, ID = intellectual disability, IS = internalizing symptoms, LSD = language and/or speech disorders.

The prevalence of ID, ADHD, LSD, and ES is increasing in patients with mutations closer to the 3′ end of the gene. Nonspecific distribution pattern of prevalence was observed for ASD, IS, and epilepsy between the patients’ groups. The highest prevalence for all comorbidities was found in patients with 3′end mutations (Supplementary Table S2; Figure 3).

The analysis of prevalence distribution between the patients’ groups reached statistical significance for ID (*p* < 0.001) and internalizing symptoms (*p* = 0.046).

### 3.3. Functional Versus Structural Comparison and Study of Dp140 5′UTR Variants

A total of 203 patients were selected for structural versus functional analysis: 72 (35.4%) patients in groups A’ and A*, 22 (10.8%) in group B’, 53 (26.1%) in group B*, 82 (40.4%) in group C’, 43 (21.1%) in group C*, 9 (4.4%) in groups D’ and D*, 8 (3.9%) in group Y, and 18 (8.9%) in groups E’ and E*.

In this cohort of 203 patients, 66 (32.5%) presented ID, 50 (24.6%) had ADHD, 17 (8.4%) had ASD, 49 (24.1%) had language and/or speech disorders, 15 (7.4%) had externalizing and 6 (2.9%) had internalizing symptoms; 12 (5.9%) patients were diagnosed with epilepsy.

**Figure 4:**
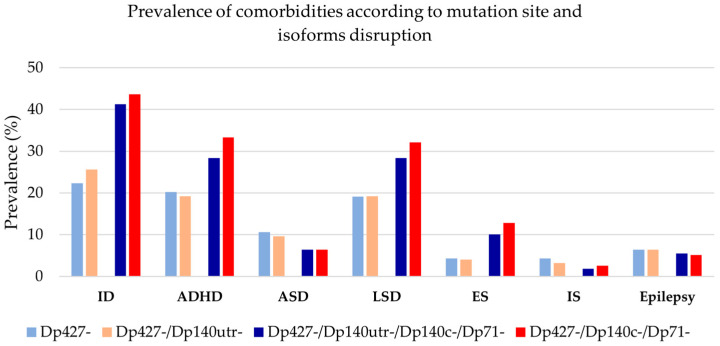
Prevalence of neuropsychiatric comorbidities for the cohort of 203 patients according to the structural classification (blue) and functional classification (red) of mutations. In patients with distal mutations, the functional classification yields higher prevalences of ID, ADHD, LSD, ES, and IS than the structural one, supporting the relevance of brain isoform-based grouping. Light blue: loss of Dp427 isoform (structural groups A’ + B’), dark blue: loss of all brain isoforms (structural groups C’ + D’ + E’), light red: loss of Dp427 (functional groups A* + B*), dark red: loss of all brain isoforms (functional groups C* + D* + Y + E*). ADHD = attention deficit hyperactive disorder, ASD = autism spectrum disorder, ES = externalizing symptoms, ID = intellectual disability, IS = internalizing symptoms, LSD = language and/or speech disorders, utr = untranslated region (genotype groups are detailed in Figure 2).

Different prevalence distribution between patients’ groups were obtained for the structural versus functional classifications (Figure 4, Supplementary Tables S3 and S4). The prevalence of ID, ADHD, LSD, ES, and IS in patients with distal mutations is higher in functional groups compared to the structural ones (Figure 4).

**Figure 5:**
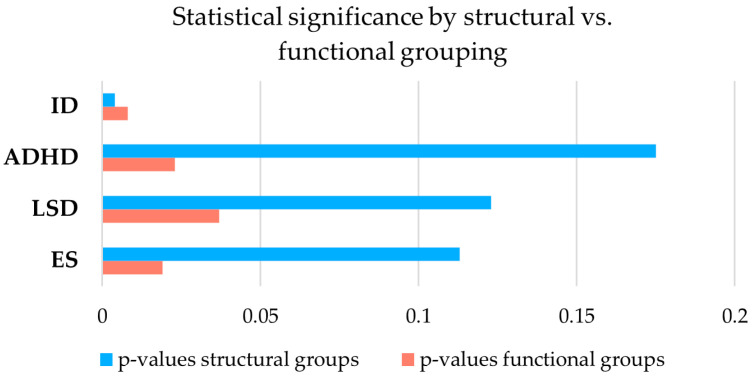
*p*-values reflecting differences in the prevalence of four comorbidities across genotype groups according to structural (blue) and functional classification (red) in the cohort of 203 patients. The figure illustrates that except for ID, no significant prevalence differences for ADHD, LSD, and ES are observed between groups in the structural classification compared to the functional one, suggesting that the isoform-based classification is more informative for neuropsychiatric risk. ADHD = attention deficit hyperactive disorder, ES = externalizing symptoms, ID = intellectual disability, LSD = language and/or speech disorders.

ID is the symptom that reached statistical significance for both types of classifications (structural: *p* = 0.004; functional: *p* = 0.008). The prevalence distribution of ADHD, LSD, and ES is statistically significant in the functional classification (*p* = 0.023, *p* = 0.037, *p* = 0.019) (Figure 5, Supplementary Tables S3 and S4).

### 3.4. Clustering of Neurological and Psychiatric Comorbidities

We determined the clustering of neurological and psychiatric comorbidities in our patients in relation to the site of the mutation. Most of the patients (143/264, 54.2%) presented at least one symptom and almost one third of patients (78/264, 29.5%) had at least two symptoms (Supplementary Table S5). Across the cohort, the maximum number of comorbidities per patient was five, which was observed in only one individual. The relationship between the number of symptoms and the genotype groups reached statistical significance (*p* = 0.027).

**Figure 6:**
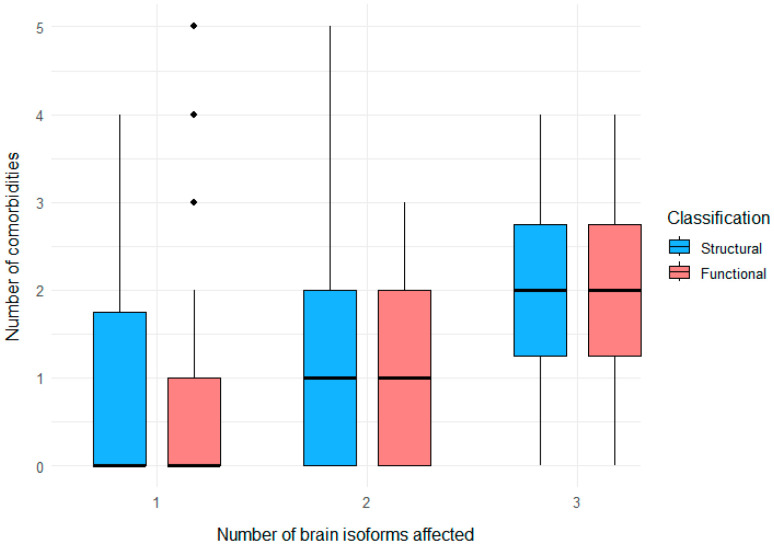
Relationship between cumulative brain dystrophin isoform loss and neuropsychiatric comorbidity burden for the cohort of 203 patients. Boxplots show the distribution of the total number of neuropsychiatric comorbidities per patient according to the number of affected brain isoforms (1, 2, or 3). Structural groups are shown in blue and functional groups in red. Boxes indicate the median and interquartile range (IQR), whiskers extend to 1.5 × IQR, and dots represent outliers. Patients with two and three affected brain isoforms have higher median comorbidity counts than those with only one affected isoform, in line with the significant positive Spearman correlations observed.

In the subset of 203 patients for whom both functional and structural classifications were available, we quantified the relationship between cumulative brain dystrophin isoform loss (1 = Dp427-; 2 = Dp427-/Dp140-; 3 = Dp427-/Dp140-/Dp71-) and the number of neuropsychiatric comorbidities per patient (range 0–5) (Figure 6). Using the functional classification, Spearman’s rank correlation showed a weak but statistically significant positive association between the number of affected brain isoforms and the number of comorbidities (*ρ* = 0.28, *p* < 0.001). The corresponding analysis based on the structural classification also revealed a significant correlation (*ρ* = 0.24, *p* < 0.001).

11 of the 14 patients with epilepsy (78.57%) presented at least one NDD symptom and 9/14 (64.2%) were diagnosed with ID. 129 from the 250 patients without epilepsy (51.6%) associated NDDs and 73/250 (29.2%) presented ID (Supplementary Table S1).

The correlation between neurological and psychiatric comorbidities was studied. A significant association was found between the following neuropsychiatric comorbidities: a. ID and ADHD (*p* = 0.009), ASD (*p* < 0.001), language and speech disorders (*p* < 0.001), and epilepsy (*p* = 0.006); b. ADHD and ASD (*p* < 0.001), language and speech disorders (*p* = 0.011), and ES (*p* < 0.001); c. ASD and language and speech disorders (*p* < 0.001); d. ES and IS (*p* = 0.012) (Supplementary Table S6).

### 3.5. Genotype–Phenotype Correlations

The phenotype in 28 groups of patients (2 to 6 patients/group) having the same variant per group was studied. The same phenotype was found in patients from 7 mutational groups. In another 7 groups, at least two patients had the same phenotype within each group. The 14 other groups included patients with either overlapping or different phenotype features (Supplementary Table S7).

Among the 11 pairs of siblings, in 7 pairs, the brothers shared the same phenotype, 2 pairs had one of the siblings presenting one more symptom, one pair had a sibling with two more symptoms and one pair of brothers presented different phenotypes (one with ID and the other presenting no symptoms) (Supplementary Table S7).

## 4. Discussion

It is now well recognized that DMD patients are at increased risk of developing neurological and psychiatric comorbidities. Consequently, the management of these symptoms is now integrated into the standard of care for these patients [34].

In the studied cohort of 264 patients, there is a high prevalence of intellectual disability, ADHD, language and/or speech disorders, and ASD. Similar percentages resulted from analyzing the reduced cohort of 203 patients. These results are concordant with previous studies reporting in DMD boys a prevalence ranging between 19 and 35% for ID [3,4,18,28], 24–39% for language and speech disorders [28,40], 11.7–32% for ADHD [24,25,41,42], and 3.79–21% for ASD [18,25,26,27,41,42].

A high prevalence of NDDs was found in patients from group A: 25% for ID, 18.1% for ADHD, 12.5% for ASD, and 18.1% for language and speech disorders as published in previous studies [18,28,42]. This suggests that disruption of the full-length Dp427 isoform is enough to develop neuropsychiatric comorbidities which could be explained by its high expression in in the cerebral cortex, hippocampus, amygdala, and cerebellum. Besides its structural function in central synapses, Dp427 clusters with GABAA receptors at inhibitory synapses and can intensify defensive behavior by affecting the amygdala GABAergic transmission [43,44].

Our analysis established that the prevalence of ID, ADHD, language and speech disorders increases when more dystrophin isoforms are affected, except for ASD. Similar results have been reported, supporting that additional loss of Dp140 and Dp71 isoforms in patients with variants closer to the 3′ end is associated with a higher prevalence of these comorbidities [18,19,20,28,40,45,46]. However, there is one study reporting comparable findings for ASD in patients with mutations upstream of exon 44 [41]. The involvement of Dp140 in NDDs has been linked to its high expression in glial cells during fetal life as well as in adult human brain in cerebral cortex, hippocampus, amygdala, and cerebellum [46,47]. *Doorenweerd* et al. reported that the cellular component associated with Dp140 is the main axon and the isoform is co-expressed with genes involved in early developmental processes such as neuron differentiation, neuron projection morphogenesis, axonal guidance, and transcription factor activity [47]. Therefore, the role of Dp140 in ND comorbidities could be explained by both the timing of its expression as well as its cellular and macroscopic localization. This role is further supported by the altered white matter microstructure [48] and the disrupted structural connectome [49] found on brain MRIs of patients lacking Dp140 isoform.

ID’s prevalence was much higher in patients from groups E (81%) and E’/E* (77.8%) compared to the other groups. This is supported by Dp71 ubiquitous expression in the fetal and adult brain [47,50], where it is involved in clustering glutamate receptors at postsynaptic densities of hippocampal neurons [51]. Additionally, it was found that Dp71 interacts with the water (AQP4) and potassium (Kir4.1) channels [43,52] in glial cells. Therefore, loss of Dp71 could lead to impaired neuronal development, due to its role in synaptic structure and function [43,51], and to altered transmembrane water permeability at the blood–brain barrier [50,51].

The prevalence of ES was 6.1% and IS—2.7%. These results are lower compared to other studies reporting 15–30% for ES [18,28] and 20–25% for IS [18,28]. The low incidence may be related to missing data from the clinic visit notes (on paper records only), even though patients received the proper care for these comorbidities.

The prevalence of epilepsy was 5.3% in the initial cohort of 264 patients and 5.9% in the reduced one. These results are in line with previous studies that reported the prevalence of epilepsy ranging between 3.1 and 12.3% [29,30,31]. A higher prevalence of NDDs was detected in patients presenting epilepsy compared to those without a diagnostic of epilepsy. These results are comparable with data reported by Hendriksen et al. [32] who proposed a triangular relationship between DMD, epilepsy, and NDDs. We are first to report a statistically significant association between epilepsy and ID (*p* = 0.006) in DMD patients, which was not detected in similar studies [32].

As a particular finding, two patients with epilepsy had variants downstream of exon 63; this was not reported by other studies performed on similar cohorts [31,32]. It is known that Dp427 is co-expressed with genes involved in epilepsy [47] and its expression was higher in the brain of patients with epilepsy compared to controls [53], while no difference could be observed for Dp140 and Dp71 [54]. Therefore, the association between prevalence and cumulative isoform loss observed for NDDs might not apply for epilepsy, even though the involvement of Dp71 in epileptogenesis was previously suggested [43,51,52]. It is possible that the epilepsy identified in these two patients might be due to the loss of Dp427 and not a result of Dp140 and Dp71 disruption, since all our patients presenting epilepsy had the full-length Dp427 isoform affected. Additionally, we found no significant *p*-value and no specific distribution of epilepsy prevalence across groups, implying that there is no correlation between mutation site and epilepsy, as suggested by a recent meta-analysis [55].

Our study analyzed brain-related comorbidities according to mutation location (structural classification) versus isoforms’ disruption (functional classification) using only variants for which the protein effect could be predicted as well as distinct grouping of Dp140 5′UTR mutations. A higher prevalence was found for ID, ADHD, LSD, and ES in patients with distal mutations included in the functional groups (5′ UTR mutations excluded) compared to the structural ones (5′UTR mutations included). Even though it is known that causative variants in 5′UTR regions can affect protein expression, our results emphasize that variants in the Dp140utr region are less likely to affect Dp140 expression and function. Although similar observations were described for ID [21,56], our study is the first to report such findings for the other comorbidities found in DMD/BMD patients. Moreover, a statistically significant difference for ADHD, LSD, and ES was obtained in the functional approach compared to the structural one. These data suggest that grouping and analyzing patients’ symptoms based on the affected isoforms, and not purely by the mutation location, is more significant and should be considered for phenotype anticipation. Additionally, we compared the cognitive phenotypes of patients carrying identical variants in Dp140 5′ UTR region, together with those already reported in the literature [21,56], but no genotype–phenotype correlation could be determined.

When analyzing the clustering of comorbidities, we found a statistically significant association between the number of isoforms affected and the number of comorbidities, which is in line with previous studies [18]. These findings indicate that patients with cumulative loss of brain dystrophin isoforms tend to present a higher neuropsychiatric burden. Additionally, this pattern was captured more strongly when patients were grouped according to the functional classification, consistent with our prevalence analyses.

The study of genotype–phenotype correlations showed that neuropsychiatric features, alone or cumulated, can vary greatly between individuals with identical variants and even between siblings. Some authors showed differences in cognitive function of unrelated males sharing the same mutation [57,58], while others observed high levels of correlation for Full-Scale Intelligence Quotient (FSIQ) in both related and unrelated subjects carrying the same DMD mutation [21]. Phenotypic variability could be explained by the co-expression networks between Dp427, Dp140, and Dp71 isoforms and genes involved in ID, NDDs, and epilepsy [47] as dystrophin isoforms might interact with these genes’ protein products. This may also explain the associations between specific comorbidities found in our study. Moreover, epigenetic factors, such as DNA methylation and histone modification, can lead to varying levels of gene expression in patients with identical variants, contributing to differences in phenotype and disease severity [59].

The limits of this study come from the retrospective design, the variable follow-up duration of patients, and the limits of the genetic investigations such as testing the integrity of promoters. For our structural versus functional approach, patients with unknown integrity of the Dp140 promoter were excluded; this introduced a bias in the statistical analysis, given that exons 45–55 represent a hotspot region for DMD deletions and duplications [60].

Despite the limitations, the strong points include the large number of participants, the advantage of a single-center study (unique practice protocol and homogeneous patients’ follow-up), and long-term monitoring.

Understanding the pathophysiology of DMD/BMD boys’ neuropsychiatric comorbidities is important not only for diagnosis, but also for the development of potential therapies. Recent studies showed that intracerebroventricular injection of the *mdx* mouse with exon skipping-based antisense oligonucleotides (ASOs), especially with tricycloDNA(tcDNA)-ASO, can partially restore brain dystrophin isoforms and improve some of the neuropsychiatric symptoms [61,62,63]. Moreover, tcDNA-ASO compounds were able to cross the blood–brain barrier after systemic treatment, giving the opportunity for a single drug to treat both muscle and brain phenotypes [64,65].

This is the first study that analyzes the difference between the structural-versus-functional approach on multiple neurological and psychiatric comorbidities of boys with Duchenne/Becker muscular dystrophy. To our knowledge, this is one of the largest European cohorts for which all these comorbidities were studied in association with DMD gene mutation site, and it is the first study of this kind performed on the Eastern European DMD/BMD population.

## 5. Conclusions

Dystrophin isoforms play a major role in brain development and function, yet a clear association between specific brain isoforms and neuropsychiatric profile remains to be established. Our study shows that patients with distal mutations, affecting additional brain isoforms, have not only higher prevalence but also a greater cumulative number of neuropsychiatric comorbidities than those with proximal variants. Conversely, we illustrate that Dp140 5′UTR variants may not affect Dp140 expression and appear to confer a lower neuropsychiatric risk than initially assumed from the classical, exon-based grouping. This emphasizes the importance of systematically documenting dystrophin isoform disruption in every patient to not only diagnose this pathology, but also to anticipate it from the perspective of quality patient care. In clinical practice, boys with distal mutations should receive structured and repeated neurological and psychiatric assessments, so that associated comorbidities can be detected early and multidisciplinary interventions can be initiated promptly, with the aim of limiting symptom progression and improving overall quality of life.

## Data Availability

The genetic and phenotype data presented in this study are openly available in the Leiden Open Variation Database (LOVD), DMD gene-specific database, at: https://www.LOVD.nl/DMD.

## Supplementary Materials

The following supporting information can be downloaded at: https://www.mdpi.com/article/10.3390/genes17010012/s1, Table S1: Genotype and phenotype data; Table S2: Prevalence of comorbidities according to the structural classification applied for cohort of 264 patients; Table S3: Prevalence of comorbidities according to the structural classification applied for cohort of 203 patients; Table S4. Prevalence of comorbidities according to the functional classification applied for the cohort of 203 patients; Table S5: Clustering of neurological and psychiatric comorbidities in accordance with patients genotype; Table S6: Associations between NDDs, behavioural-emotional symptoms and epilepsy; Table S7: Genotype -phenotype correlations.
