# Supplementary material for: Brain Matters in Duchenne Muscular Dystrophy: DMD Mutation Sites and Their Association with Neurological Comorbidities Through Isoform Impairment
Source: Genes (Basel). 2025 Dec 24;17(1):12. doi: 10.3390/genes17010012 (PMC12841320; doi:10.3390/genes17010012)
Supplement: Supplementary file 1 [file genes-17-00012-s001.zip › Supplementary Tables S2-S7.pdf]

Supplementary Information

Brain matters in Duchenne muscular dystrophy: DMD mutation sites and their association with neurological comorbidities through isoform impairment

Teodora Barbarii [1,2], Raluca Anca Tudorache [3,4], Dana Craiu [2,3,5] *, Elena Neagu [6], Lacramioara Aurelia Brinduse [7], Carmen Magdalena Burloiu [5], Catrinel Mihaela Iliescu [3,5], Magdalena Budisteanu [8,9,10], Ioana Minciu [3,5], Diana Gabriela Barca [2,3,5], Carmen Sandu [3,5], Oana Tarta-Arsene [3,5], Cristina Pomeran [5], Cristina Motoescu [3,5], Alice Dica [3,5], Cristina Anghelescu [11], Dana Surlica [5], Adrian Ioan Toma [12] and Niculina Butoianu [3,5]

[1] Department of Medical Genetics, "Carol Davila" University of Medicine and Pharmacy, Bucharest 020032, Romania

[2] Romanian Group for Undiagnosed Rare Diseases, Genomics Research and Development Institute, Bucharest 020021, Romania

[3] Pediatric Neurology Discipline, Department of Neurosciences, "Carol Davila" University of Medicine and Pharmacy, Bucharest 050474, Romania

[4] Université Paris Cité, Paris, France

[5] Pediatric Neurology Clinic, "Prof. Dr. Alexandru Obregia" Clinical Hospital, Expertise Center for Rare Pediatric Neurology Disorders, Bucharest 041914, Romania

[6] Dr. Nicolae Robanescu National Clinical Center for Children Neurorehabilitation, Bucharest 041408, Romania

[7] Public Health and Management Department, "Carol Davila" University of Medicine and Pharmacy, Bucharest 050463, Romania

[8] Research Department for Psychiatry, "Prof. Dr. Alexandru Obregia" Clinical Hospital, Bucharest 041914, Romania

[9] Medical Genetics Laboratory, Victor Babes National Institute of Pathology, Bucharest 050096, Romania

[10] Department of Genetics, Faculty of Medicine, "Titu Maiorescu" University, Bucharest 040051, Romania

[11] Ioana Medical Center, Bucharest, Romania

[12] Faculty of Medicine, "Titu Maiorescu" University, Bucharest 040051, Romania

*Correspondence: Dana Craiu, email: dcraiu@yahoo.com

Supplementary Table S2. Prevalence of comorbidities according to the structural classification applied for cohort of 264 patients

Prevalence of comorbidities according to mutation site						
	**A+B**(N=109) n(%)	**C+D**(N=134) n(%)	**E**(N=21) n(%)	Total (N=264) n(%)	P - Value	Chi-square/Fisher's exact test
**Neurodevelopmental features**						
Intellectual disability	25(22.9)	40(29.9)	17(81)	82(31.1)	<0.001	27.864
ADHD	24(22)	31(23.1)	8(38.1)	63(23.9)	0.275	2.584
ASD	10(9.2)	6(4.5)	3(14.3)	19(7.2)	0.157	3.702
Language and/or speech disorders	21(19.3)	34(25.4)	6(28.6)	61(23.1)	0.439	1.645
**Emotional behavioral problems**						
Externalizing symptoms	5(4.6)	8(6)	3(14.3)	16(6.1)	0.233	2.913
Internalizing symptoms	4(3.7)	1(0.7)	2(9.5)	7(2.7)	0.046	6.165
**Epilepsy**	7 (6.4)	5(3.7)	2(9.5)	14(5.3)	0.433	1.676

The prevalence of ID, ADHD, language and speech disorders and externalizing symptoms increases in patients with variants closer to the 3' end of the gene. The prevalence distribution of ID and IS between patients' groups is statistically significant (ID: p<0.001; IS: p=0.046). All comorbidities have the highest prevalence in patients with 3' end mutations.

Supplementary Table S3. Prevalence of comorbidities according to the structural classification applied for cohort of 203 patients

Prevalence of comorbidities according to mutation site					
	**A'+B'**(N=94) n(%)	**C'+D'+E'**(N=109) n(%)	Total(N=203) n(%)	P -Value	Chi-square/Fisher's exact test
**Neurodevelopmental features**					
Intellectual disability	21(22.3)	45(41.3)	66(32.5)	0.004	8.255
ADHD	19(20.2)	31(28.4)	50(24.6)	0.175	1.840
ASD	10(10.6)	7(6.4)	17(8.4)	0.280	1.169
Language and/or speech disorders	18(19.1)	31(28.4)	49(24.1)	0.123	2.380
**Emotional behavioral problems**					
Externalizing symptoms	4(4.3)	11(10.1)	15(7.4)	0.113	2.512
Internalizing symptoms	4(4.3)	2(1.8)	6(3)	0.310	1.031
**Epilepsy**	6(6.4)	6(5.5)	12(5.9)	0.791	0.070

The prevalence of ID, ADHD, language and speech disorders and externalizing symptoms increases in patients with variants closer to the 3' end of the gene. The prevalence distribution of ID between patients' groups is statistically significant (p=0.004).

Supplementary Table S4. Prevalence of comorbidities according to the functional classification applied for the cohort of 203 patients

Prevalence of comorbidities according to mutation site					
	**A*+B***(N=125) n(%)	**C*+D*+Y+E***(N=78) n(%)	Total(N=203) n(%)	P -Value	Chi-square/Fisher's exact test
**Neurodevelopmental features**					
Intellectual disability	32(25.6)	34(43.6)	66(32.5)	0.008	7.084
ADHD	24(19.2)	26(33.3)	50(24.6)	0.023	5.168
ASD	12(9.6)	5(6.4)	17(8.4)	0.425	0.637
Language and/or speech disorders	24(19.2)	25(32.1)	49(24.1)	0.037	4.332
**Emotional behavioral problems**					
Externalizing symptoms	5(4)	10(12.8)	15(7.4)	0.019	5.461
Internalizing symptoms	4(3.2)	2(2.6)	6(3)	0.795	0.068
**Epilepsy**	8(6.4)	4(5.1)	12(5.9)	0.709	0.140

The prevalence of ID, ADHD, language and speech disorders and externalizing symptoms increases in patients with variants closer to the 3' end of the gene. The prevalence distribution of ID, ADHD, LSD and ES between patients' groups is statistically significant (ID: p=0.008, ADHD: p=0.023, LSD: p=0.037, ES: p=0.019).

Supplementary Table S5. Clustering of neurological and psychiatric comorbidities in accordance with patients genotype

	**Mutations between exons 1-30 (A)** n (%)	**Mutations between exons 31-79 (B+C+D+E)** n (%)	**All mutations** n (%)
**Symptoms**			
0	42 (58.3)	79 (41.1)	121 (45.8)
1	11 (15.3)	54 (28.1)	65 (24.6)
2	8 (11.1)	39 (20.3)	47 (17.8)
3	7 (9.7)	15 (7.8)	22 (8.3)
4+	4 (5.6)	5 (2.6)	9 (3.4)
Total n	72	192	264

The relationship between the number of symptoms and the genotype groups is statistically significant (p-value=0.027).

Supplementary Table S6. Associations between NDDs, behavioural-emotional symptoms and epilepsy

	Intellectual disability	ADHD	ASD	Language and/or speech disorders	Externalizing symptoms	Internalizing symptoms	Epilepsy
Intellectual disability	-	0.162** 0.009	0.383** <0.001	0.273** <0.001	-0.033 0.590	-0.009 0.886	0.170** 0.006
ADHD	0.162** 0.009	-	0.325** <0.001	0.157* 0.011	0.230** <0.001	0.074 0.233	-0.014 0.826
ASD	0.383** <0.001	0.325** <0.001	-	0.230** <0.001	-0.071 0.251	-0.046 0.456	0.065 0.292
Language and/or speech disorders	0.273** <0.001	0.157* 0.011	0.230** <0.001	-	-0.026 0.670	0.077 0.210	0.031 0.619
Externalizing symptoms	-0.033 0.590	0.230** <0.001	-0.071 0.251	-0.026 0.670	-	0.156* 0.012	-0.060 0.330
Internalizing symptoms	-0.009 0.886	0.074 0.233	-0.046 0.456	0.077 0.210	0.156* 0.012	-	-0.039 0.526
Epilepsy	0.170** 0.006	-0.014 0.826	0.065 0.292	0.031 0.619	-0.060 0.330	-0.039 0.526	-

A significant association ($p<0.05$) was found between the following neuropsychiatric comorbidities: a. ID and ADHD, ASD, language and speech disorders, epilepsy; b. ADHD and ASD, language and speech disorders, externalizing symptoms; c. ASD and language and speech disorders; d. externalizing symptoms and internalizing symptoms

Supplementary Table S7. Genotype-phenotype correlations

Mutation	Group	Patients	ID	ADHD	ASD	L/S D	ES	IS	Epilepsy
del01-02	A,A’,A*	p1, p2	-	-	-	-	-	-	-
dup02	A,A’,A*	p1, p2, p3	-	-	-	-	-	-	-
del03-16	A A’,A*	p1, p2	-	-	-	-	-	-	-
del08-17	A,A’,A*	p1, p2	-	-	-	-	-	-	-
del46-48	C,C’,B*	p1, p2	-	-	-	-	-	-	-
del46-50	C,C’,B*	p1, p2, p3	+	-	-	-	-	-	-
del49-50	C,C’,B*	p1, p2	-	-	-	-	-	-	-
E02, c.40_41del; p.(Glu14Argfs*17)	A,A’,A*	p1, p2	-	-	-	-	-	-	-
		p3	-	-	-	+	-	-	-
del45-52	C,C’,C*	p1, p2	-	-	-	-	-	-	-
		p3	-	-	-	+	-	-	-
		p4	-	+	-	-	-	-	-
del48-49	C,C’,B*	p1, p2, p3, p4	-	-	-	-	-	-	-
		p5	-	-	-	+	-	-	-
del50	C,C’,B*	p1, p2	+	-	-	-	-	-	-
		p3, p4	-	-	-	-	-	-	-
del50-52	C,C’,C*	p1, p2	-	+	-	-	+	-	-
		p3	+	+	-	-	-	-	-
		p4	-	-	-	-	-	-	-
del51	C,C’,C*	p1, p2,p3	-	-	-	-	-	-	-
		p4	+	+	-	-	+	-	-
		p5	-	+	-	-	+	-	-
		p6	-	+	+	+	-	-	-
E76, c.10801C>T; p.(Gln3601*)	E,E’,E*	p1, p2	+	-	-	-	-	-	+
		p3	+	+	-	-	-	-	-
del03	A,A’,A*	p1	-	-	-	-	-	-	-
		p2	+	+	+	-	-	-	-
del08-09	A,A’,A*	p1	+	-	-	+	-	-	-
		p2	-	-	-	-	-	+	-
del13-17	A,A’,A*	p1	-	-	-	-	-	-	-
		p2	+	-	-	-	-	-	-
		p3	-	+	-	-	+	-	-
dup18	A,A’,A*	p1	-	-	-	-	-	-	-
		p2	+	-	-	+	-	-	-
del42-43	B,B’,B*	p1	-	-	-	-	-	-	-
		p2	-	+	-	-	-	-	-
E43, c.6283C>T; p.(Arg2095*)	B,B’,B*	p1	+	+	-	+	-	-	-
		p2	-	-	-	+	-	+	-
del45-54	C,C’,C*	p1	+	-	-	-	-	-	-
		p2	+	-	-	+	-	-	-
del46-47	C,C’,B*	p1	+	+	+	-	-	-	-
		p2	-	+	-	+	-	-	-
del48-50	C,C’,B*	p1	-	-	-	-	-	-	-
		p2	-	-	-	-	+	-	-
		p3	+	-	-	+	-	-	-
		p4	-	+	-	-	-	-	-
		b1	+	+	+	+	-	-	+
		b2	+	+	-	-	-	-	+
del48-52	C,C’,C*	p1	+	-	-	+	-	-	-
		p2	-	+	-	+	-	-	-
		p3	+	-	-	-	-	-	+
		p4	-	-	-	+	-	-	-
del52	C,C’,C*	p1	+	+	-	-	-	-	-
		p2	-	+	-	+	-	-	-
		p3	-	-	-	-	-	-	-
E70, c.10141C>T; p.(Arg3381*)	E,E’,E*	p1	+	-	-	+	+	-	-
		p2	+	-	+	-	-	-	-

Mutation	Group	Patients	ID	ADHD	ASD	L/S D	ES	IS	Epilepsy
E70, c.10171C>T; p.(Arg3391*)	E,E',E*	p1	-	+	-	+	-	-	-
		p2	+	+	-	-	-	-	-
		p3	+	-	-	-	-	-	-
I70, c.10223+1G>A	E,E',E*	p1	+	+	-	+	-	-	-
		p2	+	-	-	-	-	-	-
E08, c.830del p.(Gln277Argfs*6)	A,A',A*	b1,b2	-	-	-	-	-	-	-
del07-17	A, A',A*	b1,b2	-	-	-	-	-	-	-
del33-34	B,B',B*	b1,b2	-	-	-	-	-	-	-
del41-44	B	b1,b2	-	+	-	-	-	-	-
del45-47	C	b1,b2	-	-	-	-	-	-	-
del52-54	C,C',C*	b1,b2	+	-	-	+	-	-	-
E60, c.9028dupT; p.(Ser3010Phefs*4)	D,D',D*	b1,b2	+	-	-	-	-	-	-
del03-41	B,B',B*	b1	-	+	-	+	-	-	-
		b2	-	-	-	+	-	-	-
del53-55	C,C',Y	b1	+	-	-	+	-	-	-
		b2	+	+	-	+	-	-	-
dup05-06	A, A',A*	b1	+	-	-	-	-	-	-
		b2	-	-	-	-	-	-	-

Abbreviations: A,A',B,B',C,C',D,D',E,E'=structural groups; A*,B*,C*,D*,E*,Y=functional groups; ADHD=attention deficit hyperactive disorder; ASD=autism spectrum disorder; b=brother; del=deletion; dup=duplication; E=exon; ES=externalizing symptoms; I=intron; ID=intellectual disability; IS=internalizing symptoms; L/S D= language and/or speech disorders; p=patient.+=present; -=absent.
